# Retraction: MicroRNA-208a-3p functions as an oncogene in colorectal cancer by targeting PDCD4

**DOI:** 10.1042/BSR-2018-1598_RET

**Published:** 2022-12-22

**Keywords:** Colorectal cancer, micrRNA-208-3p, PDCD4

This article is being retracted from *Bioscience Reports* at the request of the corresponding author following receipt of a notification from a reader, alerting the Editorial Office to similarities between figures from this paper with those from other papers by unrelated authors. These include between the Figure 7A PDCD4 western blots and the Caprin1 blots from Figure 5B of Yu et al. 2020 (doi: 10.1007/s13205-020-02433-9), the Figure 3A HCT116 miR-208a-3p inhibitor panel and the miR-302c mimics panel of Figure 3A from Wu et al. 2019 (doi: 10.1042/BSR20190421), and the Figure 2D HCT116 Inhibitor NC panel and the Figure 2G mimics NC panel of the previous Wu et al. 2019 paper. The Editorial Office also noted a similarity in the western blots of Figure 4, where the PDCD4/HCT116 band appeared to be horizontally flipped for the PDCD4/SW480 bands. The co-authors have requested that their names be removed from the authorship list and have stated that they were unaware of, and never authorised, the article. The corresponding author cooperated with our investigation and provided the raw flow cytometry and cell sample data for Figures 3A, 5E and 2D. However, given the concerns with the original data and that the authorship dispute was not resolved, the Editorial Board and Editor in Chief stand by the retraction. The corresponding author agrees to the retraction.

